# Comparison of trastuzumab deruxtecan and sacituzumab govitecan in HER2-negative metastatic breast cancer: a large real-world data analysis

**DOI:** 10.1186/s13058-025-02094-7

**Published:** 2025-08-11

**Authors:** George W. Jr. Sledge, Joanne Xiu, Jeffrey Peter Solzak, Jennifer Ribeiro, Reshma L. Mahtani, Maryam B. Lustberg, Matthew J. Oberley, Milan Radovich, David Spetzler

**Affiliations:** 1 Caris Life Sciences, 4610 S 44th Pl, Phoenix, AZ 85040 USA; 2 Baptist Health Miami Cancer Institute, Miami, FL 33176 USA; 3 Yale School of Medicine, New Haven, CT 06510 USA

**Keywords:** Trastuzumab-deruxtecan; sacituzumab govitecan, Antibody-drug conjugates; metastatic breast cancer, HER2, Real-world data

## Abstract

**Background:**

Trastuzumab deruxtecan (T-DXd) and sacituzumab govitecan (SG) are antibody-drug conjugates (ADCs) increasingly used in HER2-negative breast cancer. We hypothesized that treatment benefit would vary across HER2-null, HER2-ultra-low, and HER2-low subgroups of HER2-negative breast cancer patients. We also aimed to study the clinical impact of different sequencing of the two ADCs.

**Methods:**

We analyzed a large, real-world cohort of 4,030 HER2-negative breast cancer specimens from patients treated with T-DXd or SG. Tumors underwent molecular profiling including HER2 status (IHC, CISH) and hormone receptor (HR) status (IHC) at Caris Life Sciences (Phoenix, AZ, USA). Real-world clinical data were obtained from insurance claims and analyzed by Cox proportional hazards.

**Results:**

HER2-low, HER2-ultra-low, and HER2-null cohorts treated with T-DXd had decreasing time-on-treatment (TOT; 4.8, 4.1, and 3.5 mo., respectively, *P* < 0.001), while HER2 status had little impact on SG TOT (3.0, 2.8, and 3.4 mo., respectively). Patients with HR+/HER2-negative tumors showed longer TOT when treated with T-DXd only (*n* = 1,049) than with SG-only (*n* = 453), even in the HER2-null subset (*P* < 0.001). In all HER2-negative patients treated with both ADCs, T-DXd-first (*n* = 547) or SG-first (*n* = 432) showed no cumulative TOT difference (10.4 vs. 10.8 mo., *P* = 0.356); however, the HER2-null subset showed preference for SG-first and this was restricted to the HR- subset [TOT: 11.7 vs. 7.4 mo., hazard ratio = 0.478 (95% CI: 0.333–0.685), *P* < 0.0001; OS: 19.7 vs. 11.8 mo., hazard ratio = 0.478 (95% CI: 0.303–0.756)].

**Conclusions:**

Analysis of this large real-world dataset allowed interrogation of T-DXd and SG benefit and treatment sequencing across HER2-negative subsets, providing important clinical insights into two widely used ADCs in breast cancer. We demonstrate improved relative outcomes associated with T-DXd in HR+ tumors across all HER2-negative subgroups and comparable benefit of the two ADCs in TNBC. Sequencing preference was seen in HR−/HER2-null patients only, favoring SG-first for TOT and OS. These findings warrant further validation in independent cohorts.

**Supplementary Information:**

The online version contains supplementary material available at 10.1186/s13058-025-02094-7.

## Background

Antibody-drug conjugates (ADCs) have transformed the treatment of metastatic breast cancer (MBC), with an expanding repertoire of cell surface targets and payloads. Accordingly, questions have arisen regarding their use in a treatment landscape that may include multiple active ADCs [1]. Trastuzumab deruxtecan (T-DXd) is a HER2-targeting ADC that was initially approved for HER2+ (IHC 3 + or 2 + and ISH amplified) MBC [2, 3]. With the enhancement of precision in HER2 categorization to include HER2-low and HER2-ultra-low groups [4], T-DXd has since gained approval in these subsets in various settings [5–8]. Similarly, sacituzumab govitecan (SG), a TROP2-directed ADC, was initially approved for triple-negative breast cancer (TNBC) but subsequently has been approved for hormone receptor (HR)+ MBC [9–13]. Originally distinct therapeutic entities, T-DXd and SG now have overlapping FDA-approved clinical indications. Furthermore, another TROP2-targeting ADC, datopotamab-deruxtecan (Dato-DXd), was recently approved for HR+, HER2-negative MBC, which is anticipated to further increase the therapeutic intersections within the field [14, 15].

The approvals for T-DXd and SG were based on several phase II and III trials: DESTINY-Breast-01, DESTINY-Breast-04, DESTINY-Breast-06, ASCENT, and TROPiCS-02 [2, 5, 6, 9, 10]. DESTINY-Breast-01 established T-DXd as preferred over chemotherapy for patients with pre-treated HER2 + MBC. DESTINY-Breast-04 then demonstrated the benefit of T-DXd over chemotherapy in patients with pre-treated HER2-low MBC; benefits were observed in HR+ patients and the whole population, although a majority of patients in the study were HR+. The DESTINY-Breast-06 trial expanded the benefit of T-DXd over chemotherapy to patients with HR+/HER2-low or HER2-ultra-low MBC who had received one or more lines of endocrine therapy. For SG, the ASCENT trial proved its efficacy over chemotherapy in relapsed or refractory metastatic TNBC, while the TROPiCs-02 trial established the benefit of SG over chemotherapy in pre-treated, endocrine-resistant HR+/HER2-negative MBC.

Because of some overlapping clinical indications for T-DXd and SG, both ADCs are often given sequentially in eligible patients. Although patients may experience cross-resistance due to T-DXd and SG containing a similar topoisomerase I inhibitor drug payload, there is some evidence of clinical benefit from this approach [1, 16–18]. However, limited data exist to support an optimal treatment sequence across different subgroups of patients [17, 19]. Instead, sequencing decisions are primarily inferred from the results of clinical trials as described above [20]. While well-designed phase III randomized controlled trials remain the gold standard to answer questions regarding treatment selection and sequence for different subsets of patients, extensive and robust real-world evidence has shown increasing power in providing provide insight into important clinical problems [20, 21].

We hypothesized that treatment benefit from T-DXd and SG would vary across groups of patients according to HER2-negative status (HER2-null, HER2-ultra-low, HER2-low) and hormone receptor (HR) status, and that outcomes may differ according to treatment sequence of the two ADCs. We focused on the HER2-negative population, as it is highly unlikely that SG, a non-HER2-targeting ADC, would be given prior to HER2-targeting drugs for patients with HER2+ tumors. To test this hypothesis, we used a large real-world database of tumors with comprehensive tumor profiling information which included immunohistochemistry (IHC) and chromogenic in-situ hybridization (CISH) data linked to patient outcomes. We aimed to reveal important insights regarding the relative benefit and optimal treatment sequence of these ADCs in different subsets of patients, which can be applied clinically for treatment selection.

## Methods

### Patient data collection

Breast tumor specimens from patients that received T-DXd and/or SG and underwent comprehensive tumor profiling at Caris Life Sciences (Phoenix, AZ, USA) were included in the study. Clinical and demographic characteristics of the cohort are detailed in Table 1 and Supplementary Table S1. Real-world clinical data were obtained from insurance claims, which encompass detailed records of health services, including prescribed medications, procedures performed, and established diagnoses. In patients treated with a single ADC, time-on-treatment (TOT) was determined as the interval from the initiation to the conclusion of that treatment, while in patients treated with both ADCs, TOT2 was determined from the start of treatment with the first ADC to the end of treatment with the second ADC. TOT was considered as the primary outcome analysis, since it more accurately reflects drug-specific treatment response and mitigates the impact of patient-to-patient differences including treatment setting. Overall survival (OS) was analyzed as a secondary outcome and was defined as the period from ADC treatment initiation to the date of the patient’s last known clinical activity. In cases with no insurance claims for a period exceeding 100 days, it was inferred that the patient had deceased. Conversely, patients with a documented clinical activity within 100 days prior to the latest data update were censored in the analysis. Kaplan-Meier survival estimates were generated for cohorts defined by molecular characteristics. Hazard ratios were computed utilizing the Cox proportional hazards model, and significant differences in survival times were assessed with the log-rank test, where *P* < 0.05 was considered significant.

**Table 1:** Patient characteristics

		Single ADC-treated			Both ADCs			
		T-DXd only, *N* (%)	SG only, *N* (%)	*P*-value	T-DXd before SG, *N* (%)	SG before T-DXd, *N* (%)	*P*-value	Total, *N* (%)
**Her2 Category**	**HER2-null**	318 (22)	1218 (67)	***< 0.0001***	127 (30)	151 (42)	***0.0021***	1815 (45)
	**HER2-ultra-low**	336 (23)	316 (17)		103 (25)	83 (23)		838 (21)
	**HER2-low**	789 (55)	273 (15)		190 (45)	126 (35)		1380 (34)
**Age**	**Median Age**	60	58	0.1	57.5	58	0.1	58
	**Interquartile Range**	51–68	48–67		50–66	47–65		49–67
**Gender**	**Female**	1427 (99)	1799 (100)	***0.04***	416 (99)	355 (99)	0.8	3997 (99)
**Race**	**Asian or Pacific Islander**	62 (4)	50 (3)	***< 0.0001***	21 (5)	13 (4)	***< 0.0001***	146 (4)
	**Black or African American**	175 (12)	343 (19)		56 (13)	64 (18)		638 (16)
	**White**	861 (60)	900 (50)		239 (57)	167 (46)		2167 (54)
	**Other**	47 (3)	74 (4)		2 (0)	18 (5)		141 (3)
	**Unknown**	298 (21)	440 (24)		102 (24)	98 (27)		938 (23)
**Ethnicity**	**Hispanic or Latino**	101 (7.0)	165 (5.9)	***< 0.01***	29 (7)	31 (9)	0.6	326 (8)
	**Not Hispanic or Latino**	1065 (74)	1224 (69)		292 (70)	251 (70)		2832 (70)
	**Unknown**	277 (19)	418 (23)		99 (24)	78 (22)		872 (22)
**Specimen Site**	**Primary/Local**	433 (30)	726 (40)	***< 0.0001***	128 (30)	139 (39)	***< 0.001***	1426 (35)
	**Non-Visceral**	461 (32)	588 (33)		118 (28)	125 (35)		1292 (32)
	**Visceral**	529 (37)	464 (26)		170 (40)	92 (26)		1255 (31)
	**Unclear/Other**	20 (1.4)	29 (1.6)		4 (1)	4 (1)		57 (1)
	**Total**	**1443**	**1807**		**420**	**360**		**4030**

Significant *P*-values are bolded and italicized

### HER2, Estrogen receptor (ER), and progesterone receptor (PR) status

Immunohistochemistry (IHC) was performed on full formalin-fixed paraffin-embedded (FFPE) sections of glass slides. Slides were stained using automated staining techniques, per the manufacturer’s instructions, and were optimized and validated per CLIA/CAP and ISO requirements. Staining was scored for intensity (0 = no staining; 1 + = weak staining; 2 + = moderate staining; 3 + = strong staining) and staining percentage (0-100%) by board-certified oncologic pathologists. Antibody used for HER2 was VENTANA^®^ Pathway anti-HER2/neu RxDx [4B5; Roche (Ventana), Tucson, AZ, USA]. IHC was used in combination with chromogenic in-situ hybridization (CISH; INFORM HER-2 Dual ISH DNA Probe Cocktail) to determine HER2 amplification status, as outlined in Wolff et al. [22]. HER2 groups were established as follows:

- HER2-positive: IHC 3 + with > 10% of cells positive (excluded from analysis).
- HER2-low: IHC 1 + with > 10% of cells positive or IHC 2 + with > 10% of cells positive combined with a negative CISH result (< 4 HER2 signals/cell (regardless of HER2/CEP17 ratio) or HER2/CEP17 ratio < 2 with average HER2 copy number ≥ 4 but < 6 signals/cell).
- HER2-ultra-low: IHC 0 with faint staining in ≤ 10% of cells.
- HER2-null: IHC 0 with no visible staining.

Additionally, the presence of ER and PR was determined by IHC using the CONFIRM Estrogen Receptor (SP1) antibody and CONFIRM Progesterone Receptor (1E2) antibody [Roche (Ventana)], with a positivity threshold of ≥ 1% for both.

## Results

### Patient characteristics and treatment distribution

A total of 4,030 breast tumors categorized as classic HER2-negative from patients treated with either T-DXd or SG were included in our study. Based on recently developed further categorization of HER2 status, 45% (*n* = 1,815) were completely devoid of HER2 protein expression (HER2-null), while 55% expressed HER2 protein, among which 34% were HER2-low (*n* = 1,380) and 21% HER2-ultra-low (*n* = 838). Notably, the vast majority of patients received only one ADC in their treatment course (81%, *n* = 3,250) while a smaller subset (19%, *n* = 780) were treated with both agents (Table 1).

The median age of patients was comparable across treatment groups. Reflecting the greater prevalence of TNBC in Black women [23], there was a higher percentage of Black patients treated with SG only compared to T-DXd only (19% vs. 12%; *P* < 0.0001) and SG first compared to T-DXd first (18% vs. 13%; *P* < 0.0001). Patients treated with SG only or SG first were more likely (by 14% and 13%, respectively) to have biopsies derived from primary sites than visceral metastases, while patients treated with T-DXd only or T-DXd first were more likely (by 7% and 10%, respectively) to have biopsies derived from visceral metastases than primary sites (*P* < 0.001; Table 1).

Among 1,807 patients treated with SG only, a majority had HER2-null tumors (67%, *n* = 1,218), with smaller percentages of HER2-ultra-low (17%, *n* = 316) and HER2-low (15%, *n* = 273; *P* < 0.0001). Conversely, among 1,443 tumors from patients treated with T-DXd only, a majority were HER2-low (55%, *n* = 789), with smaller percentages of HER2-ultra-low (23%, *n* = 336) and HER2-null (22%, *n* = 318; *P* < 0.0001). Similarly, when examining patients treated with SG before T-DXd, a plurality was HER2-null (42%, *n* = 151/360), while a plurality of patients treated with T-DXd before SG had HER2-low tumors (45%, *n* = 190/420; *P* = 0.002; Table 1). These differences reflect real-world clinicians’ treatment choices for HER2-negative tumors based on HER2 status, and the preferential use of T-DXd and SG in HER2-low and HER2-null tumors, respectively.

### Correlation of HER2 status with T-DXd but not SG TOT

**Fig. 1:**
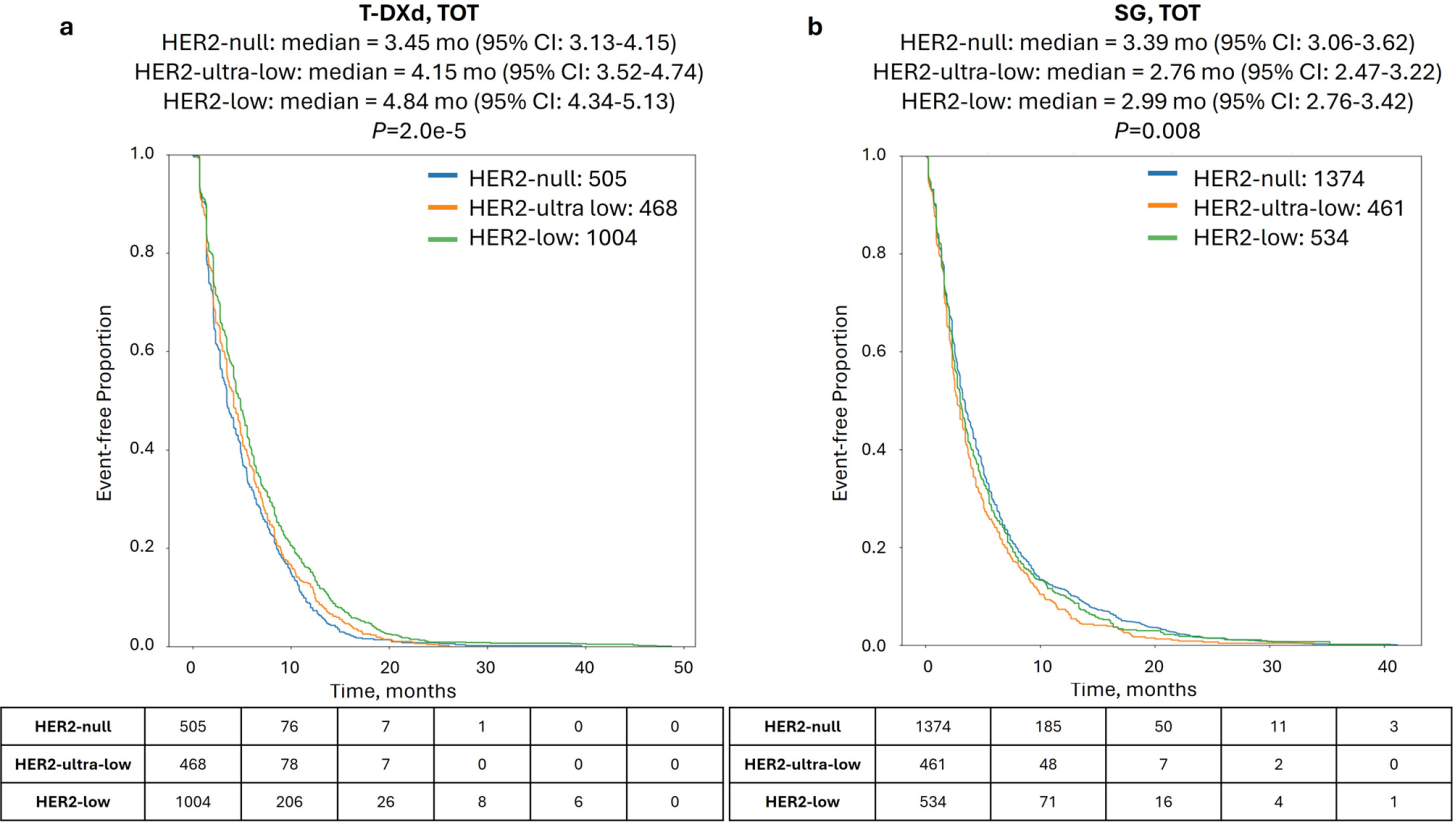
Time-on-treatment (TOT) with T-DXd and SG according to HER2 status. Kaplan-Meier survival analysis of TOT with T-DXd **(a)** and SG **(b)** in HER2-null (blue lines), HER2-ultra-low (orange lines), and HER2-low (green lines)

When investigating patient outcomes in cohorts stratified by HER2 status, HER2-low, HER2-ultra-low, and HER2-null cohorts treated with T-DXd had decreasing time-on-treatment (TOT; 4.84, 4.15, and 3.45 mo., *P* = 2.0e-5; Fig. 1a). Conversely, while SG TOT significantly differed according to HER2 status (*P* = 0.008), there was no decreasing trend observed (2.99, 2.76, and 3.39 mo. for HER2-low, HER2-ultra-low, and HER2-null, respectively; Fig. 1b).

### T-DXd shows superior TOT compared to SG in HR+ tumors

**Fig. 2:**
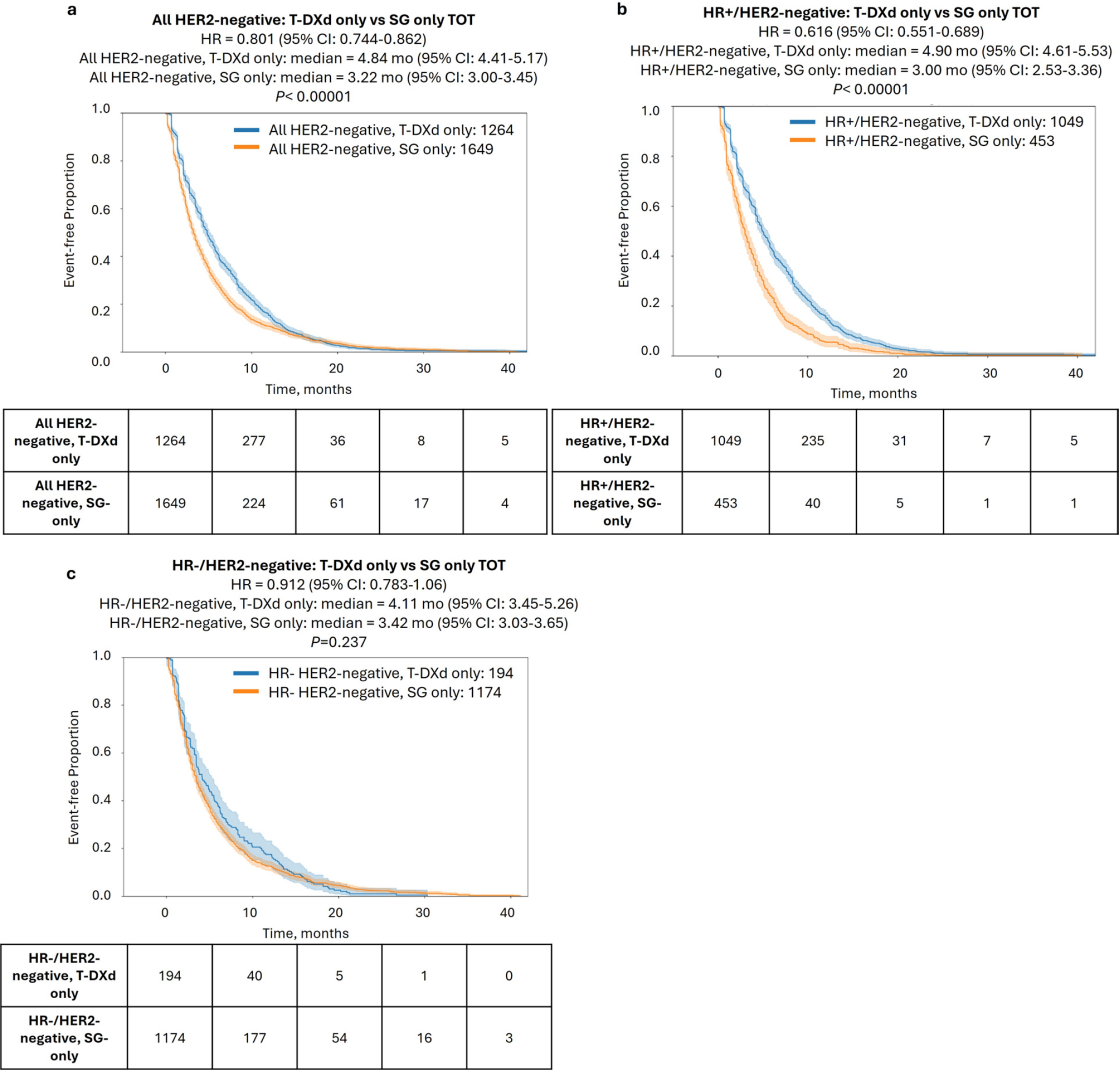
Time-on-treatment (TOT) with T-DXd only vs. SG only. Kaplan-Meier curves show TOT with T-DXd only vs. SG only in all HER2-negative patients **(a)**, HR+/HER2-negative patients **(b)**, and HR−/HER2-negative patients **(c)**

We next compared TOT with T-DXd and SG in all HER2-negative patients treated with one ADC only. Notably, a significantly longer TOT was seen for T-DXd compared to SG [4.8 vs. 3.2 mo., hazard ratio = 0.801, 95% CI%: 0.744–0.862, *P* < 0.00001; Fig. 2a], which also held true in multivariate analysis considering other treatments received, specimen site, and *BRCA1/2* mutation status (Supplementary Table S2). Interestingly, when stratifying by patients’ HR status, the effect was prominent in the HR+ subset [4.9 vs. 3.0 mo., hazard ratio = 0.616 (95% CI: 0.551–0.689), *P* < 0.0001; Fig. 2b) but was not significant in the HR- subset [4.1 vs. 3.4 mo., hazard ratio = 0.912 (95% CI: 0.783–1.063), *P* = 0.237; Fig. 2c].

The striking TOT difference between HR+ and HR− patients prompted further investigation into TOT differences in HER2-negative categories (Table 2). Perhaps expectedly, we observed a significantly prolonged TOT with T-DXd compared to SG in both HER2-ultra-low [4.8 vs. 3.0 mo., hazard ratio = 0.71 (95% CI: 0.59–0.83), *P* < 0.001] and HER2-low cases [ 4.9 vs. 3.5 mo., hazard ratio = 0.83 (95% CI: 0.83-1.0), *P* = 0.011]. More unexpectedly, T-DXd showed a longer TOT than SG even in the HER2-null group [4.7 vs. 3.4 mo., hazard ratio = 0.91 (95% CI: 0.77-1.0)], although this did not reach the level of significance (*P* = 0.053). We next stratified by HR status. As we observed in the full HER2-negative cohort, the benefit of T-DXd over SG was restricted to HR+ tumors and was highly significant for all HER2-negative subsets including HER2-null tumors (*P* < 0.001; Table 2).

**Table 2:** Time-on-treatment (TOT) with T-DXd only and SG only in HER2 stratified cohorts

	All				HR+				HR−			
HER2 category	TOT (T-DXd; SG), months	*N* (T-DXd; SG)	Hazard Ratio (95% CI)	*P*-value	TOT (T-DXd; SG), months	*N* (T-DXd; SG)	Hazard Ratio (95% CI)	*P*-value	TOT (T-DXd; SG), months	*N* (T-DXd; SG)	HazardRatio (95% CI)	*P*-value
**HER2-null**	4.7; 3.4	262; 1116	0.91 (0.77-1)	0.053	4,8; 3.0	209; 277	0.67 (0.56–0.83)	***< 0.001***	4.6; 3.5	48; 822	0.91 (0.71–1.3)	0.6
**HER2-ultra-low**	4.8; 3.0	295; 289	1.4 (0.59–0.83)	***< 0.001***	5.1; 2.5	245; 99	0.56 (0.45–0.71)	***< 0.001***	3.2; 3.0	46; 188	0.91 (0.63–1.3)	0.4
**HER2-low**	4.9; 3.5	707; 244	1.2 (0.83-1)	***0.011***	5.1; 3.1	595; 77	0.67 (0.53–0.83)	***< 0.001***	4.2; 3.9	100; 164	0.91 (0.77–1.3)	0.7

Significant *P*-values are bolded and italicized

The exclusive benefit of T-DXd over SG in HR+ tumors led us to investigate whether HR status was a determining factor of T-DXd benefit. Interestingly, no significant effect was seen when comparing HR+ vs. HR− tumors across various HER2 categories for T-DXd (Supplemental Fig. S1a-b); in contrast, shorter TOT (but not OS) with SG was seen in HR+ tumors compared to HR− tumors [3.0 vs. 3.4 mo., hazard ratio = 1.3 (95% CI: 1.1–1.4), *P* < 0.00001)] (Supplemental Fig. S1c-d). Further analysis revealed significantly higher gene expression of *TROP2* in HR− tumors [median TPM = 86.7, (95% CI: 79–92)] compared to HR+ [median = 65.8 (95% CI: 60–76)], consistent with previous reports [26]; however, a direct association of *TROP2* expression and SG outcome was not seen (Supplementary Fig. S1e-f), suggesting that the improved benefit of SG in HR− tumors is not directly related to *TROP2* expression.

### Sequencing of T-DXd and SG impacts outcomes in HR−/HER2-null only

**Fig. 3:**
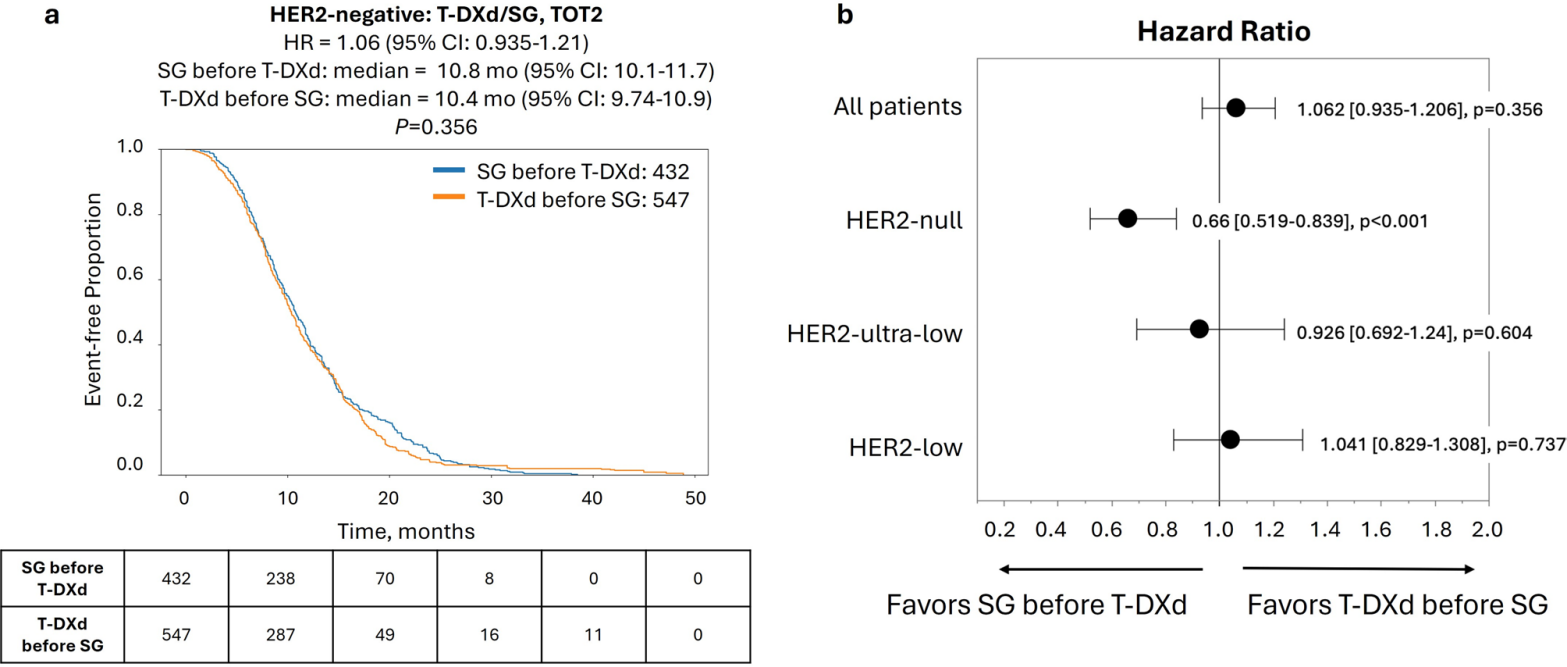
Time-on-treatment from start of first ADC to end of second ADC (TOT2) for patients in the full HER2-negative cohort according to treatment sequence of T-DXd and SG. **(a)** Kaplan-Meier curve shows median TOT2 of patients treated with SG before T-DXd (blue lines) vs. T-DXd before SG (orange lines). **(b)** Forest plot of hazard ratios for TOT2 with T-DXd before SG vs. SG before T-DXd in patient cohorts according to HER2 status

**Fig. 4:**
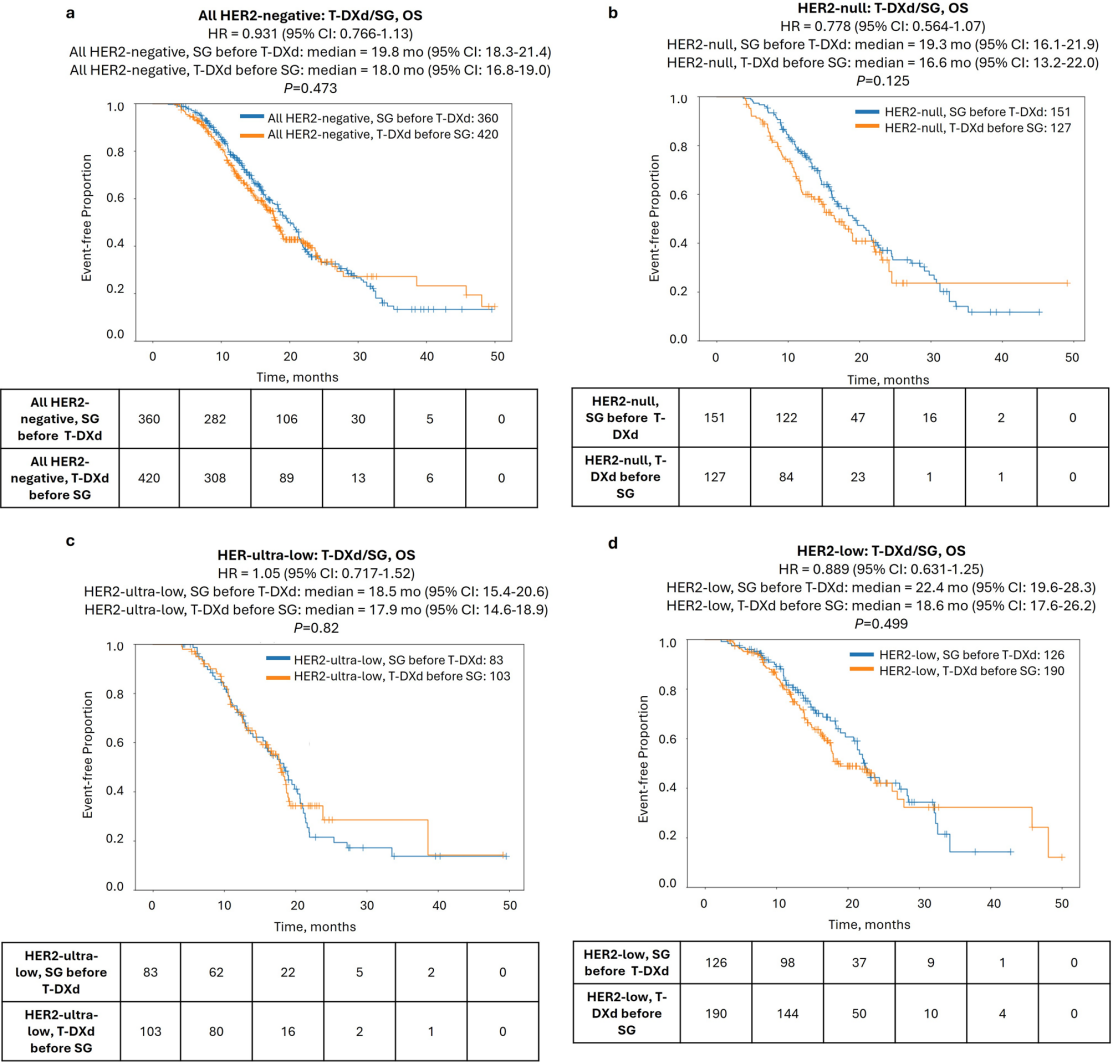
ADC-associated overall survival (OS) of patients in the HER2-negative cohort according to treatment sequence of T-DXd and SG. Kaplan-Meier curves show median OS of patients treated with T-DXd before SG (orange lines) vs. SG before T-DXd (blue lines) in the full HER2-negative cohort **(a)** and HER2-null **(b)**, HER2-ultra-low **(c)**, and HER2-low **(d)** cohorts

Finally, we examined outcomes according to the sequence of ADC treatment, comparing the interval from the start of the first ADC to the end of the second ADC (TOT2). In the full HER2-negative cohort, TOT2 was nearly identical for patients treated with T-DXd first versus SG first (Fig. 3a). When stratifying based on HER2 status, HER2-null cases showed a modest TOT2 advantage when treated with SG first compared to T-DXd first [hazard ratio = 0.66 (95% CI: 0.52–0.84), *P* < 0.001], while no difference in TOT2 was seen in the HER2-ultra-low and HER2-low groups (Fig. 3b, Supplementary Fig. S2). Similarly, no difference in OS (calculated from the start of the first ADC agent to last contact) was observed between patients treated with T-DXd first versus SG first in any of the HER2-negative subgroups (Fig. 4).

**Fig. 5:**
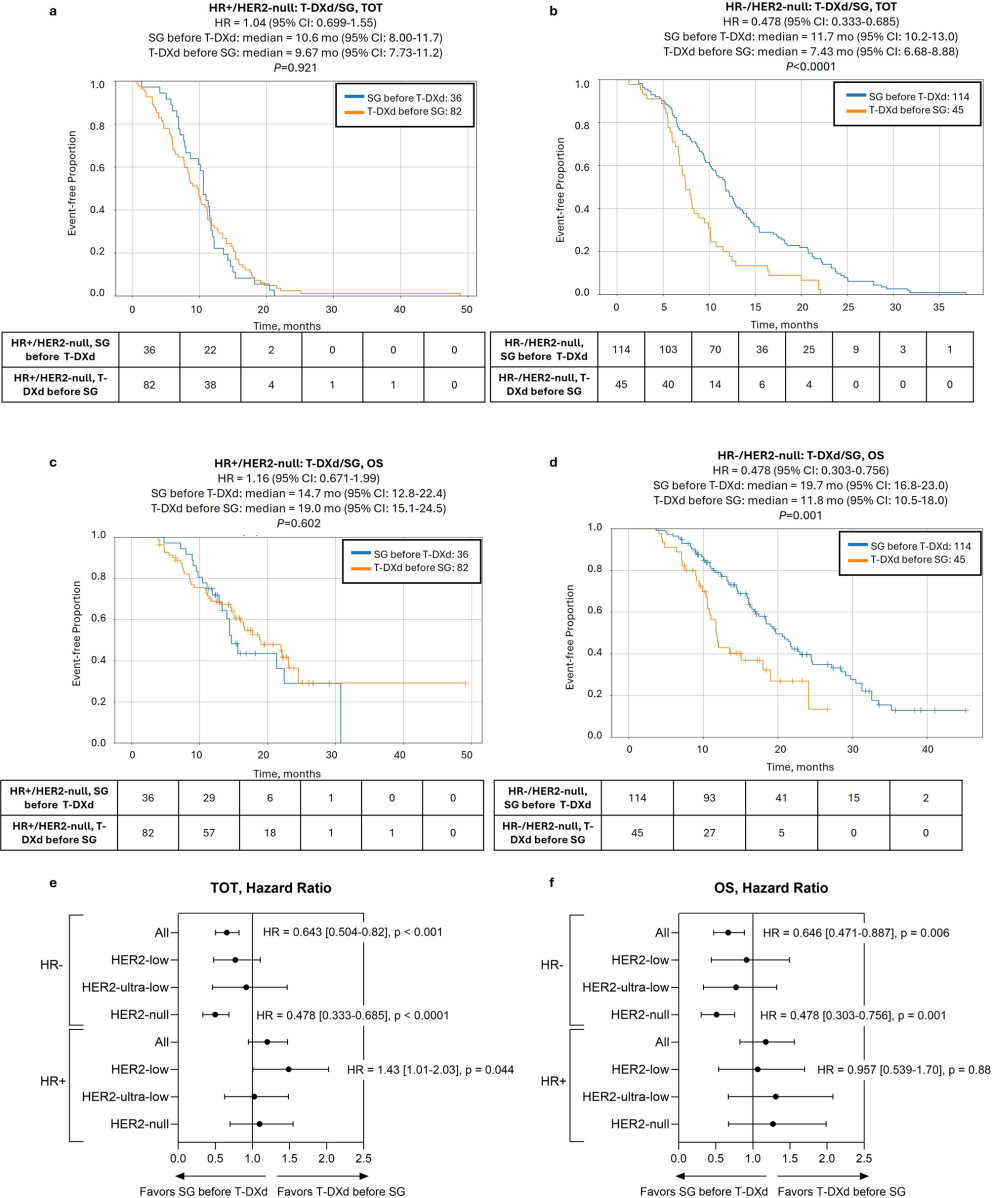
ADC sequencing-associated outcomes in patients with HER2-null tumors according to HR status. Kaplan-Meier curves show time-on-treatment from start of first ADC to end of second ADC (TOT2) **(a**,** b)** and overall survival (OS) **(c**,** d)** comparing SG before T-DXd (blue lines) vs. T-DXd before SG (orange lines) in HR+/HER2-null **(a**,** c)** and HR−/HER2-null groups **(b**,** d)**

Further stratification by HR status revealed that sequencing mainly impacted outcomes in HR−/HER2-null tumors. There was a significant prolongation of TOT2 [11.7 vs. 7.4 mo., hazard ratio = 0.478 (95% CI: 0.333–0.685), *P* < 0.0001] when patients with HR−/HER2-null tumors were treated with SG-first compared to T-DXd-first, but no differences were noted in HR+ tumors in any HER2-negative subgroup. Notably, this difference translated to a significant prolongation of OS as well [19.7 vs. 11.8 mo., hazard ratio = 0.478 (95% CI: 0.303–0.756), *P* = 0.001] (Fig. 5). Conversely, T-DXd showed some preference in HR+/HER2-low tumors, with shorter TOT2 for SG-first [8.3 vs. 11.1 mo., hazard ratio = 1.43 (95% CI: 1.01–2.03)], although the *P*-value was borderline (*P* = 0.044) and there was no difference in OS (Fig. 5, Supplementary Figs. S3-4).

## Discussion

T-DXd and SG are two active ADCs that independently improve OS in MBC patients. Though initially being limited to either classic HER2+ MBC (T-DXd) or TNBC (SG), the presence of their cell surface targets outside of these initial subsets led to their expanded use following demonstration of therapeutic efficacy in several pivotal trials trials [2, 3, 5–13]. Similarly, the expansion of what physicians consider HER2+ for therapeutic purposes (HER2-low and HER2-ultra-low tumors) [4] has led to therapeutic overlap, wherein patients with similar breast cancer subsets may receive both agents. This overlap allowed us to investigate relative efficacy and sequencing for these drugs.

We first examined the question of relative efficacy when these drugs are used as single agents, using the measure of TOT obtained from insurance claims. In alignment with results from the phase II DAISY trial [24], HER2 status (null, ultra-low and low) was correlated with median T-DXd TOT. Again unsurprisingly, HER2 status was not correlated with SG outcomes, since SG targets TROP2 rather than HER2. Of note, TOT durations in our study were generally shorter than progression-free survival durations observed in the pivotal trials, likely due to TOT being impacted by real-world scenarios such as heterogeneous performance status and drug tolerability.

Comparing T-DXd and SG as single agents, patients who received T-DXd but not SG had longer median TOT compared to SG, but this difference was restricted to HR+ patients. Furthermore, the prominence of T-DXd’s benefit over SG in HR+ tumors across multiple HER2 categories—including HER2-null tumors—is noteworthy. It has been suggested that the benefit of T-DXd in HER-ultra-low or HER2-null tumors could derive from very low levels of HER2 allowing uptake of T-DXd, from the presence of free payload following cleavage of the T-DXd linker, or from HER2-independent mechanisms [24]. While it is well known that HER2 status impacts the extent of benefit of T-DXd [24], a direct comparison between HR+ vs. HR− tumors has not been reported. Our study provides suggestive data in a real-world setting that T-DXd may outperform SG in HR+ tumors, while the two agents appear to confer comparable benefit in HR− tumors. The exact mechanism for this benefit difference remains to be determined, but our data points to SG, not T-DXd, underlying the effect. We initially considered it plausible that this difference could be partly driven by higher TROP2 levels leading to improved therapeutic benefit of SG in HR− tumors, but we observed no difference in SG outcomes based on* TROP2* expression. Therefore, further research is needed to understand various direct or indirect factors leading to improved SG benefit in HR− tumors compared to HR+.

Data on optimal ADC sequencing has been limited. One small, retrospective study indicated longer OS from start of ADC1 in patients with HER2-low disease treated with SG-first compared to T-DXd-first, but this study was not designed to analyze this outcome [19]. In our cohort, we found that the sequence of the ADCs had minimal impact on outcomes; however, an exception was seen in the HR−/HER2-null subgroup, where SG-first sequencing was associated with a significant TOT2 and OS advantage. Conversely, we found some preference for T-DXd-first in patients with HR+/HER2-low tumors; however, given the modest difference in TOT2 and no difference in OS, this sequencing preference may not be clinically meaningful. Patients with HR− tumors that were HER2-low or HER2-ultra-low showed comparable responses to SG and T-DXd and no sequencing preference, indicating that clinical decisions for these groups can be made based on other factors. Overall, these results both support and offer refinement to National Comprehensive Cancer Network (NCCN) guidelines on the use of T-DXd and SG in MBC [25]. Supplementary Fig. S5 summarizes the clinical benefit and sequencing preference for these two ADCs that is supported by the results of our study.

Our study has several strengths, including its large sample size (*N* = 4,033) and comprehensive molecular profiling, which enabled granular stratification by HER2 and HR status. The use of real-world data from insurance claims provides a snapshot of clinical practice on these new ADC agents, with great generalizability to the clinic. The limitations of this study relate primarily to the limitations of real-world data: as shown in Table 1, the use of SG and T-DXd is non-random across major breast cancer subsets, with SG-only patients being preferentially HER2-null and T-DXd-only patients being far more commonly HER2-low. Prior and intervening therapies are also potential confounding factors and must be further explored to understand how treatment setting affects ADC sequencing. However, we observed similar intervening time intervals between ADC1 and ADC2 in T-DXd-only treated patients (1.5 months, IQR: 0.9–3.9 months) and SG-only treated patients (1.6 months, IQR: 0.7–4.55 months), suggesting minimal differences in intervening therapies between groups in our cohort. Furthermore, prior studies have shown that ADC effectiveness largely depends on the patient’s disease biology rather than intervening therapies [17, 19], suggesting that intervening therapies are not likely to be major confounders for our results.

Another limitation is that the timing of both initial and subsequent FDA approvals for T-DXd and SG differed, particularly regarding their respective expansions that resulted in eventual therapeutic overlap. Physicians who use these agents sequentially may choose a particular sequence for particular perceived rationales regarding sequence efficacy or toxicity. Prospective clinical trials such as the ongoing TRADE-DXd trial (NCT06533826), the ENCORE trial (NCT06774027), and the SERIES trial (NCT06263543) can complement and validate our real-world findings. TRADE-DXd aims to analyze the benefit of treatment sequence of T-DXd and datopotamab-DXd in HER2-low tumors. ENCORE (NCT06774027), a prospective registry trial, aims to determine the benefit of sequential ADCs in HER2-negative MBC. SERIES specifically aims to investigate the efficacy of SG following T-DXd in patients with HR+/HER2-low MBC.

## Conclusions

In conclusion, our analysis demonstrates that T-DXd offers a significantly prolonged TOT compared to SG in HR+ MBC across HER2-negative categories, while both agents perform comparably in HR− tumors. Sequencing preferences emerge only in HR− tumors lacking any HER2 expression (HR−/HER2-null), favoring SG-first as is shown by both TOT and OS; while outcomes remain largely unaffected by sequence across other groups. Our study provides important insight into personalized treatment strategies in HER2-negative MBC and warrants further validation in independent datasets or in randomized controlled trials.

## Supplementary Information

Below is the link to the electronic supplementary material.

Supplementary Material 1

## Data Availability

The deidentified sequencing data are owned by Caris Life Sciences and cannot be publicly shared due to the data usage agreement in place. These data will be made available to researchers for replication and verification purposes through our letter of intent process, which are generally fulfilled with 6 months. For more information on how to access this data, please contact Joanne Xiu at jxiu@carisls.com.

## Abbreviations

ADC: Antibody-drug conjugate
CI: Confidence interval
ER: Estrogen receptor
HR: Hormone receptor
IQR: Interquartile range
OS: Overall survival
PR: Progesterone receptor
SG: Sacituzumab govitecan
T-DXd: Trastuzumab deruxtecan
TOT: Time-on-treatment
TNBC: Triple-negative breast cancer
