# Supplementary material for: Comparison of trastuzumab deruxtecan and sacituzumab govitecan in HER2-negative metastatic breast cancer: a large real-world data analysis
Source: Breast Cancer Res. 2025 Aug 11;27:144. doi: 10.1186/s13058-025-02094-7 (PMC12341277; doi:10.1186/s13058-025-02094-7)
Supplement: Supplementary file 1 — Supplementary Material 1 [file 13058_2025_2094_MOESM1_ESM.docx]

**Comparison of Trastuzumab Deruxtecan and Sacituzumab Govitecan in HER2-Negative Metastatic Breast Cancer: A Large Real-World Data Analysis**

**Authors:** George W. Sledge Jr.^1^, Joanne Xiu^1^, Jeffrey Peter Solzak^1^, Jennifer Ribeiro^1^, Reshma L. Mahtani^2^, Maryam B. Lustberg^3^, Matthew J. Oberley^1^, Milan Radovich^1^, David Spetzler^1^

**Affiliations:**

^1^Caris Life Sciences, 4610 S 44th Pl, Phoenix, AZ, USA 85040

^2^Baptist Health Miami Cancer Institute, Miami, FL, USA 33176

^3^Yale School of Medicine, New Haven, CT, USA 06510

**Corresponding Author:**

George W. Sledge Jr.

Caris Life Sciences

4610 S 44th Pl,

Phoenix, AZ 85040

(469) 724-6502

[gsledge@carisls.com](mailto:gsledge@carisls.com)

ORCID: 0000-0003-0297-0775

**Supplementary Table S1. Additional clinical characteristics of study cohort**

|  | **Feature** | **TDXd-only, N (%)** | **SG-only, N (%)** | ***P*-value** |
| --- | --- | --- | --- | --- |
| **Other treatments** | **TDM1** | 161 (11.2) | 17 (0.9) | <0.0001 |
|  | **Chemo** | 1219 (84.5) | 1741 (96.3) |  |
|  | **Hormone** | 1306 (90.5) | 1008 (55.8) |  |
|  | **CDK4/6 Inhibitors** | 844 (58.5) | 369 (20.4) |  |
| **BRCA mutation status** | **BRCA1/2 mt.** | 95 (6.6) | 114 (6.3) | 0.73 |
|  | **Total** | 1443 | 1807 |  |

**Supplementary Table S2. Multivariate analysis in all HER2-negative patients**

| **Comparison** | **Adjusted Hazard Ratio** | **HR 95% CI** | **Adjusted *P*-value** |
| --- | --- | --- | --- |
| **TDXd-only vs. SG-only** | 0.808 | 0.738-0.885 | <0.0001 |
| **TDM1 vs. no TDM1** | 0.718 | 0.608-0.847 | <0.0001 |
| **Chemo vs. no Chemo** | 0.95 | 0.828-1.09 | 0.464 |
| **Hormone therapy vs. no hormone therapy** | 0.987 | 0.898-1.084 | 0.782 |
| **CDK4/6 inhibitor vs. no CDK4/6 inhibitor** | 1.085 | 0.990-1.189 | 0.08 |
| **Primary vs. Visceral Metastasis** | 1.006 | 0.918-1.103 | 0.898 |
| **Primary vs. Non-Visceral Metastasis** | 1.124 | 1.028-1.229 | 0.01 |
| **BRCA1/2 mt. vs. BRCA1/2 wildtype** | 0.897 | 0.773-1.041 | 0.153 |

**Supplementary Fig. S1 ADC outcomes according to TROP2 expression and HR status. (a, b)** Forest plots summarize hazard ratios (HRs) for T-DXd-only time-on-treatment (TOT) **(a)** and overall survival (OS) **(b)** in HR+ vs. HR- groups in all HER2-negative tumors and HER2-negative subsets. **(c, d)** Kaplan-Meier curves for SG-only TOT **(c)** and OS **(d)** in all HER2-negative tumors according to HR status. **(e, f)** Kaplan-Meier curves for SG-only TOT **(e)** and OS **(f)** in groups according to TROP2 (*TACSTD2*) gene expression quartiles.

**
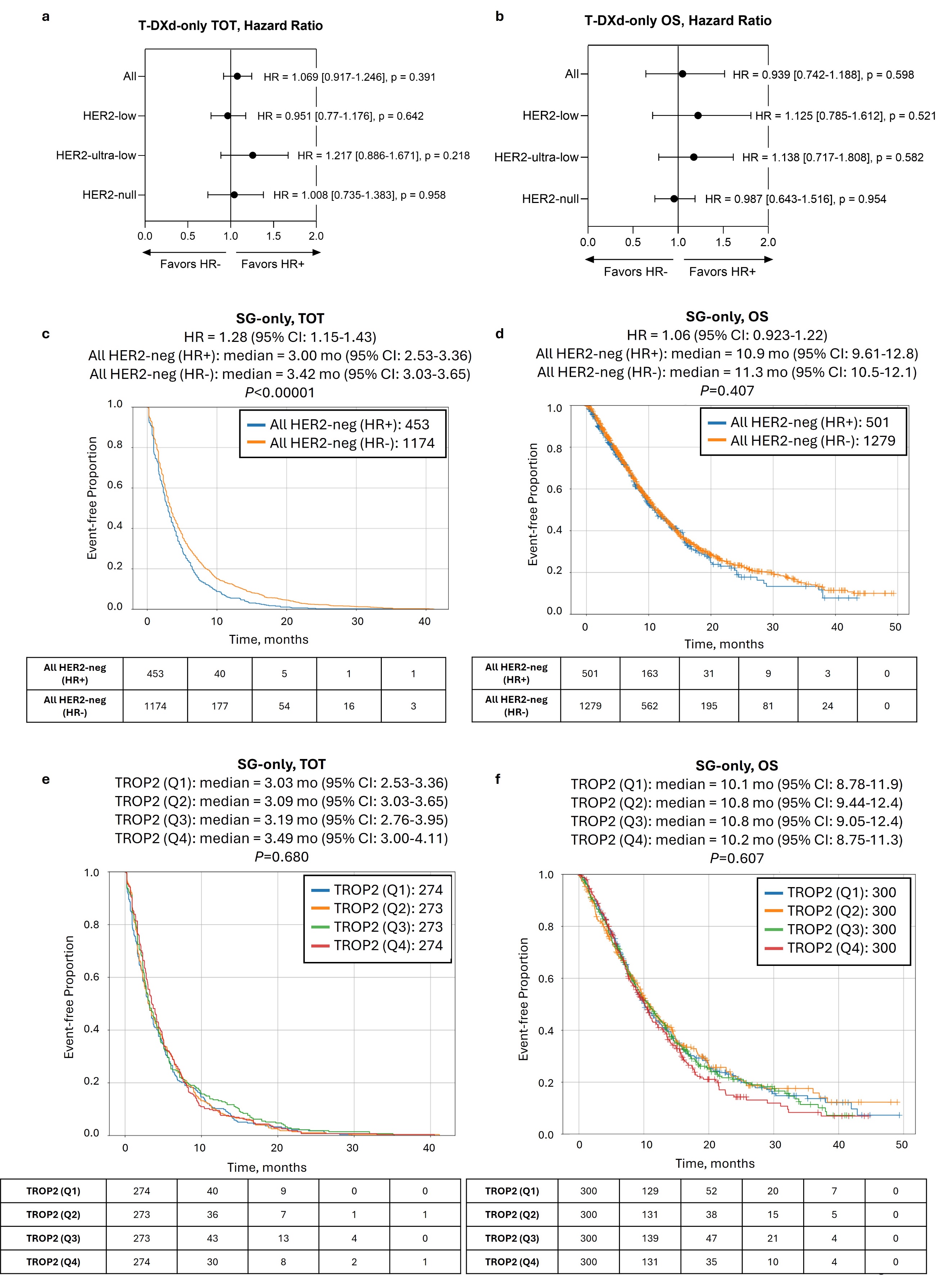
**

**Supplementary Fig. S2 Time-on-treatment from start of first ADC to end of second ADC (TOT2) for patients in HER2-negative subgroups according to treatment sequence of T-DXd and SG.** Kaplan-Meier curves show TOT2 for SG before T-DXd (blue lines) and T-DXd before SG (orange lines) in HER2-null group **(a)**, HER2-ultra-low group **(b)**, and HER2-low group **(c)**.


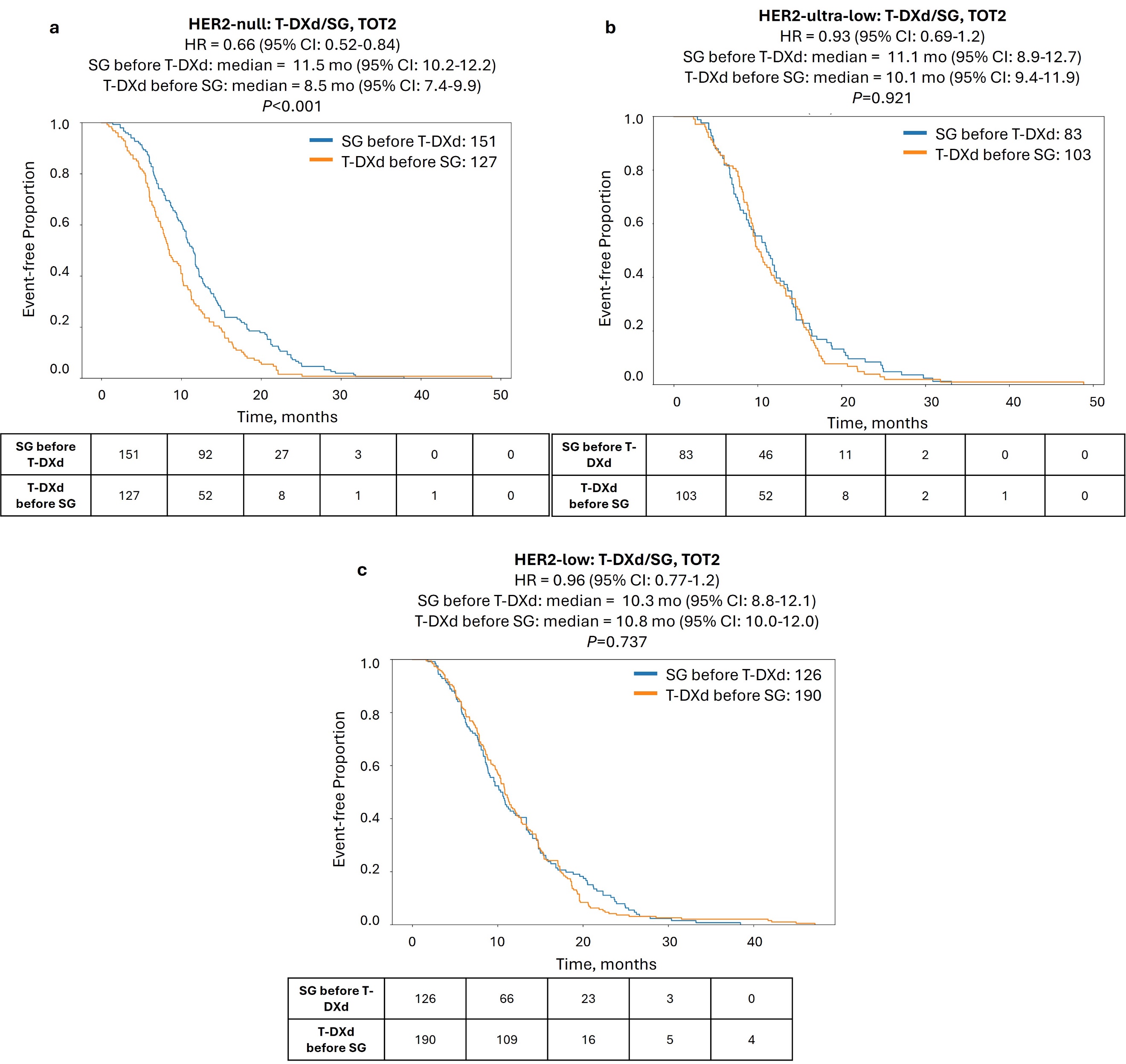


**
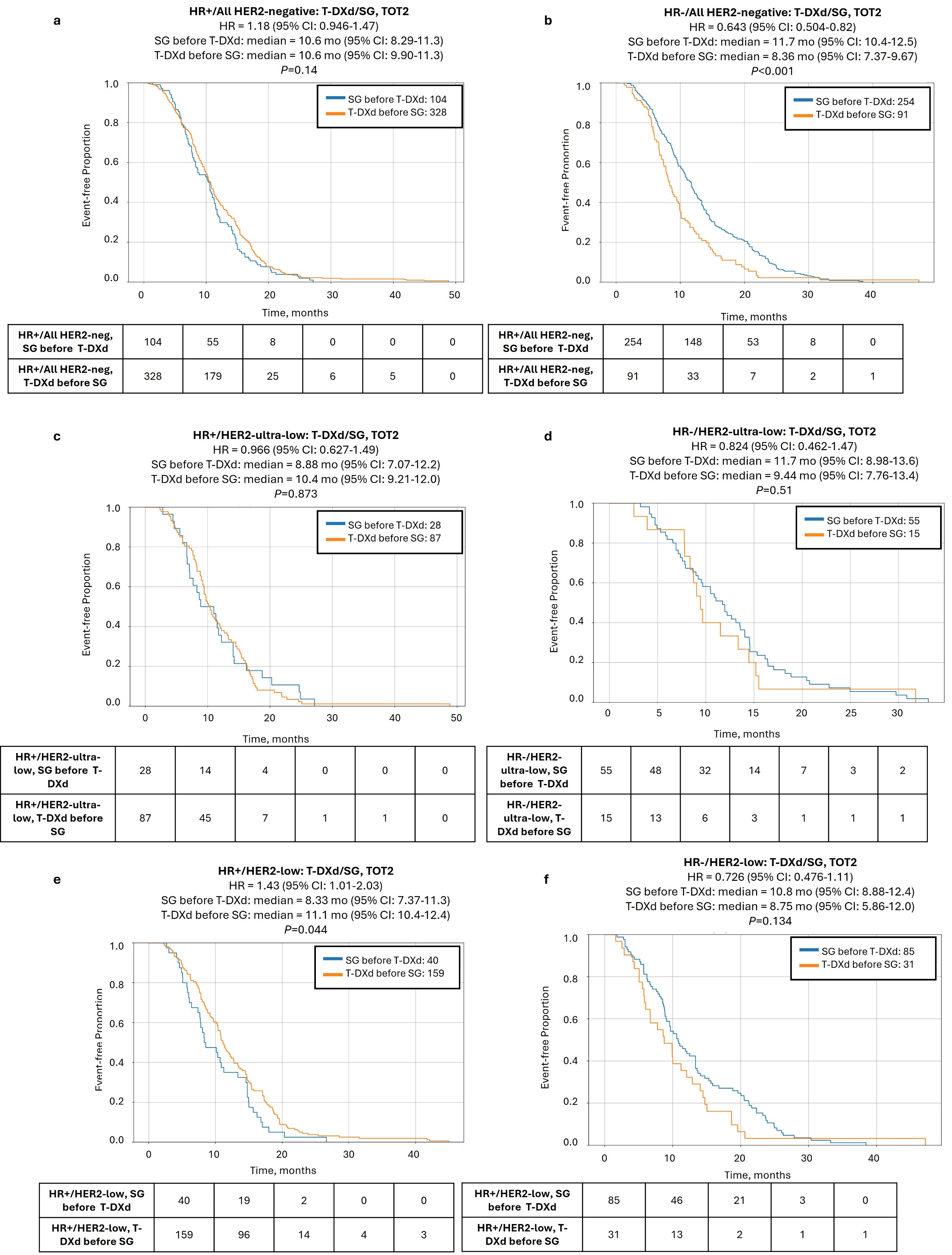
Supplementary Fig. S3 Time-on-treatment from start of first ADC to end of second ADC (TOT2) for ADC sequencing in HER2-negative subsets stratified by HR status.** Kaplan-Meier curves show TOT2 in all HER2-negative **(a, b)**, HER2-ultra-low **(c, d)**, and HER2-low tumors **(e, f)**, stratified by HR+ **(a, c, e)** and HR- tumors **(b, d, f)**.


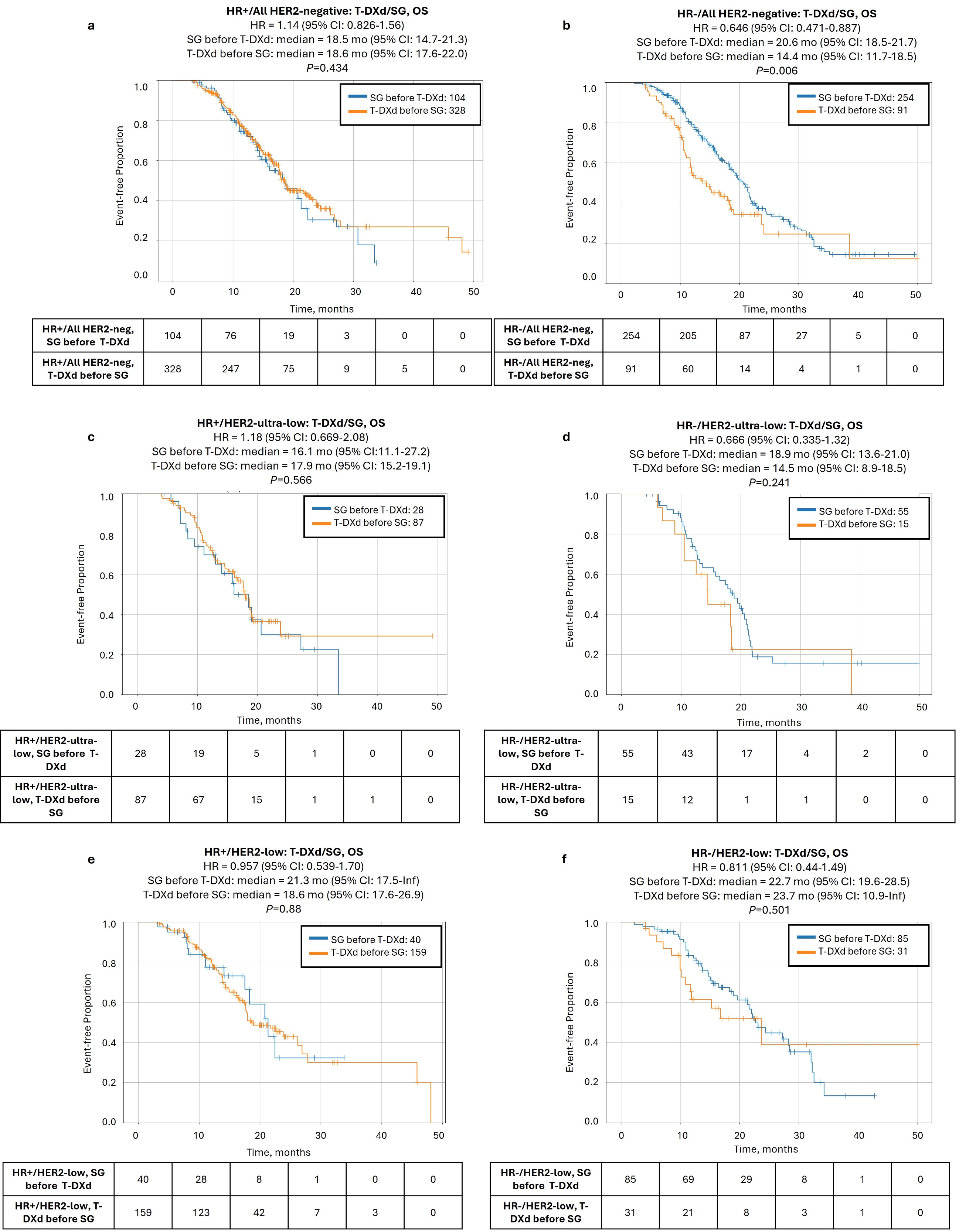
**Supplementary Fig. S4 Overall survival (OS) for ADC sequencing in HER2-negative subsets stratified by HR status.** Kaplan-Meier curves show OS from start of first ADC to last contact in all HER2-negative tumors **(a, b)**, HER2-ultra-low **(c, d)**, and HER2-low **(e, f)**, stratified by HR+ tumors **(a, c, e)** and HR- tumors **(b, d, f)**.

**Supplementary Fig. S5 Schematic depicting ADC benefit and optimal sequencing according to HR and HER2 status.** Dotted box: suggestion based on borderline improved TOT and *P*-value.

**
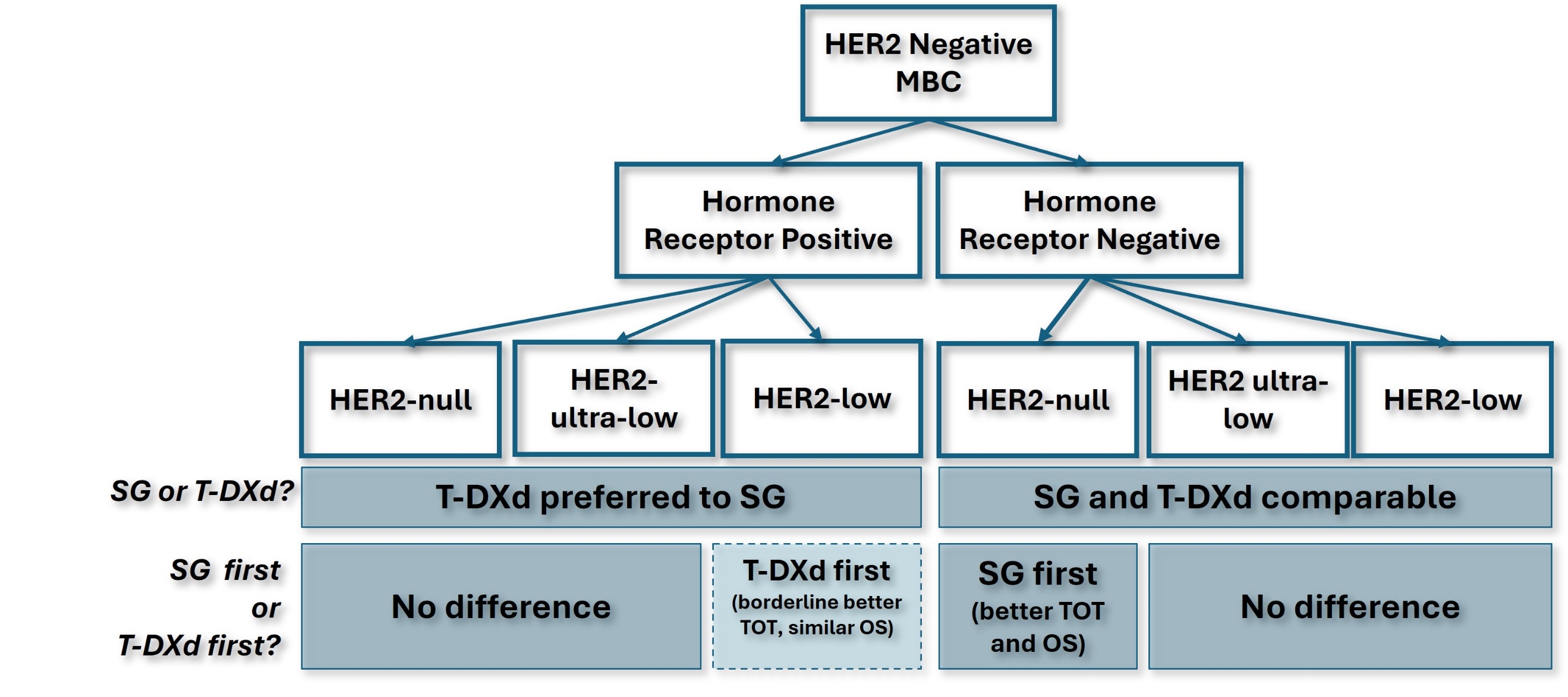
**
